# Henipavirus Neutralising Antibodies in an Isolated Island Population of African Fruit Bats

**DOI:** 10.1371/journal.pone.0030346

**Published:** 2012-01-12

**Authors:** Alison J. Peel, Kate S. Baker, Gary Crameri, Jennifer A. Barr, David T. S. Hayman, Edward Wright, Christopher C. Broder, Andrés Fernández-Loras, Anthony R. Fooks, Lin-Fa Wang, Andrew A. Cunningham, James L. N. Wood

**Affiliations:** 1 Department of Veterinary Medicine, University of Cambridge, Cambridge, United Kingdom; 2 Institute of Zoology, Zoological Society of London, Regent's Park, London, United Kingdom; 3 CSIRO Livestock Industries, Australian Animal Health Laboratory, Geelong, Victoria, Australia; 4 Animal Health and Veterinary Laboratories Agency, Wildlife Zoonoses and Vector-Borne Diseases Research Group, Department of Virology, Veterinary Laboratories Agency, Weybridge, New Haw, Addlestone, Surrey, United Kingdom; 5 Department of Biology, Colorado State University, Fort Collins, Colorado, United States of America; 6 School of Life Sciences, University of Westminster, London, United Kingdom; 7 Division of Infection and Immunity, University College London, London, United Kingdom; 8 Department of Microbiology and Immunology, Uniformed Services University, Bethesda, Maryland, United States of America; 9 National Centre for Zoonosis Research, Leahurst, Neston, South Wirral, United Kingdom; Global Viral Forecasting Initiative, United States of America

## Abstract

Isolated islands provide valuable opportunities to study the persistence of viruses in wildlife populations, including population size thresholds such as the critical community size. The straw-coloured fruit bat, *Eidolon helvum*, has been identified as a reservoir for henipaviruses (serological evidence) and Lagos bat virus (LBV; virus isolation and serological evidence) in continental Africa. Here, we sampled from a remote population of *E. helvum annobonensis* fruit bats on Annobón island in the Gulf of Guinea to investigate whether antibodies to these viruses also exist in this isolated subspecies. Henipavirus serological analyses (Luminex multiplexed binding and inhibition assays, virus neutralisation tests and western blots) and lyssavirus serological analyses (LBV: modified Fluorescent Antibody Virus Neutralisation test, LBV and Mokola virus: lentivirus pseudovirus neutralisation assay) were undertaken on 73 and 70 samples respectively. Given the isolation of fruit bats on Annobón and their lack of connectivity with other populations, it was expected that the population size on the island would be too small to allow persistence of viruses that are thought to cause acute and immunising infections. However, the presence of antibodies against henipaviruses was detected using the Luminex binding assay and confirmed using alternative assays. Neutralising antibodies to LBV were detected in one bat using both assays. We demonstrate clear evidence for exposure of multiple individuals to henipaviruses in this remote population of *E. helvum annobonensis* fruit bats on Annobón island. The situation is less clear for LBV. Seroprevalences to henipaviruses and LBV in Annobón are notably different to those in *E. helvum* in continental locations studied using the same sampling techniques and assays. Whilst cross-sectional serological studies in wildlife populations cannot provide details on viral dynamics within populations, valuable information on the presence or absence of viruses may be obtained and utilised for informing future studies.

## Introduction

Reservoir host population size and density play a critical role in the ability of a species to maintain viruses that cause acute or immunising infections, reflected through epidemiological principles such as the critical community size (CCS) and the effective reproductive number (R_eff_). The CCS is a theoretical population threshold describing the minimum number of individuals in a population required for virus persistence [1]. It is unrealistic to consider this threshold absolute; rather it should be viewed as ‘the host population size above which stochastic fadeout of a disease over a given period is less probable than not’ [2]. Typically, pathogens causing acute immunising infections require large host population sizes to maintain an adequate supply of susceptible individuals to maintain transmission [3], unless birth rates are very high. Also important in shaping pathogen transmission dynamics is host population density, via its effect on R_eff_: the expected number of secondary infections that arise from each primary infection in a partially immune population [2]. Together, these factors mean that host species which exist in large population sizes and in high densities are capable of acting as reservoirs for a greater number of viruses than smaller, low density populations [4], [5].

Of the species that fulfil these population characteristics and that live in close proximity to humans, bats have been highlighted as reservoirs of many emerging zoonotic diseases, such as SARS-like coronaviruses, henipaviruses, filoviruses and lyssaviruses [6]. In some cases, multiple potentially-zoonotic viruses have been identified in a single host species, such as the straw-coloured fruit bat (*Eidolon helvum*). This migratory, tree-roosting species, forms very large seasonal colonies across sub-Saharan Africa [7]–[9], often near large human populations [10], [11]. The timing of *E. helvum* birth pulses and migrations vary across its continental range [8], but little is known about the connectivity between populations. This species has been identified as a reservoir for henipaviruses and Lagos bat virus (LBV, genus *Lyssavirus*) in continental Africa [12]–[18]. Other viruses detected in *E. helvum* include a novel orbivirus [19] and rotavirus [20] (both via viral isolation), a coronavirus [21] (via PCR), and a filovirus [22] (via presence of antibodies), however insufficient information is available to determine whether it is an incidental or a reservoir host for these viruses. It could be hypothesised that the large asynchronous metapopulation of *E. helvum* ensures an ongoing supply of susceptible individuals for new infections, however mechanisms of viral transmission and maintenance at the population level are unknown.

**Figure 1:**
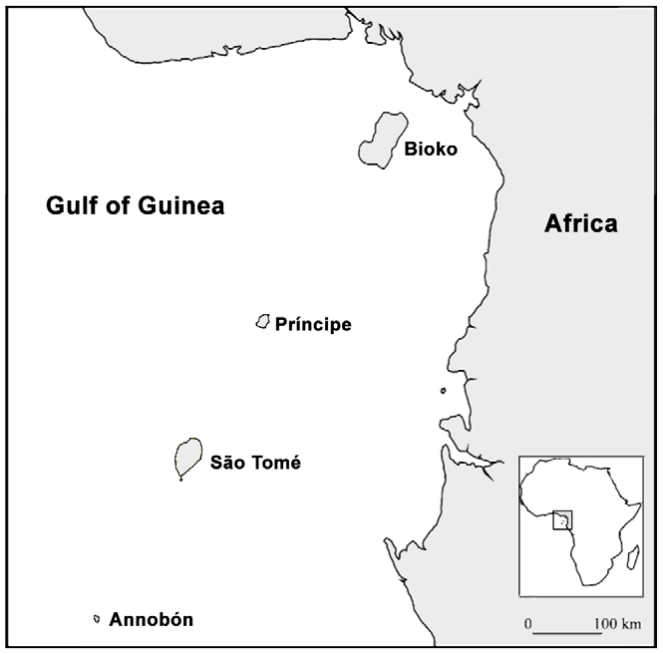
Map of the Gulf of Guinea islands indicating the location of Annobón.

In addition to its widespread continental distribution, *E. helvum* exists on a small number of off-shore islands, including those in the Gulf of Guinea: Bioko, Príncipe, São Tomé and Annobón [23] (Figure 1). Although all four islands are part of the Cameroon volcanic chain, Bioko was previously connected to the mainland via a land bridge, while Príncipe, São Tomé and Annobón formed independently 31, 13 and 4.8 million years ago, respectively [24] i.e. these latter three islands are, and always have been, isolated from the mainland and from each other. Annobón is the smallest and most isolated of these islands, with an area of just 17.5 km^2^, and lying 183 km from the nearest island and 340 km from the continent. Juste *et al*. [23] established that the *E. helvum* population on Annobón is significantly smaller in body size than populations on the nearest islands or on continental Africa. Additionally, allozyme analyses identified corresponding genetic differentiation, with the rate of gene flow between Annobón and other islands or continental populations approaching the minimum required for independent divergence by random drift [23]. In fact, Annobón's geographic isolation has resulted in sufficient genetic differentiation of *E. helvum* on the island for its designation as a separate subspecies, *E. helvum annobonensis* [23].

Here, we use serology to investigate if henipavirus or LBV infections exist in the geographically isolated population of *E. helvum annobonensis* fruit bats on Annobón island.

## Methods

### Sample collection

**Figure 2:**
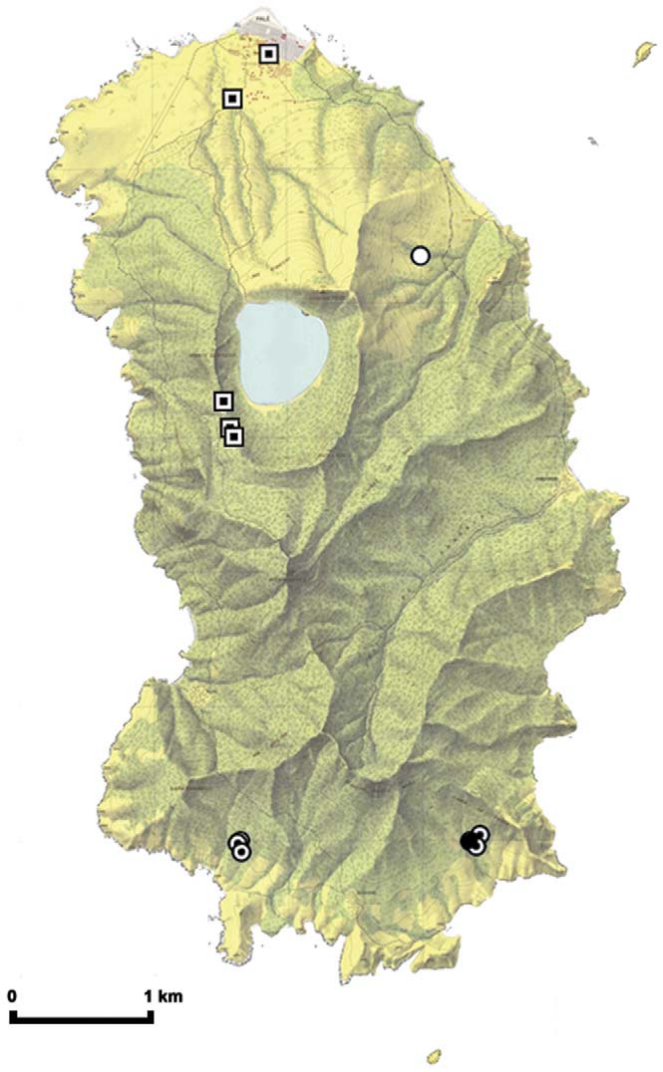
Map of Annobón indicating *Eidolon helvum* colonies and sampling sites. Key: Circles indicate colony locations (open circle: colonies were reported or used to exist in the past, but no bats were found; partially filled circle: colony observed, but not sampled; filled circle: colony observed and sampled). Squares indicate sites where *Eidolon* bats have been observed feeding at different times of the year.

The sampling protocol used was approved by the Zoological Society of London Ethics Committee (WLE/0849), and all fieldwork was approved by the Equatorial Guinea Ministry of Agriculture and Forestry. Samples were collected from one of two known *E. helvum annobonensis* colonies on Annobón (‘Adjo’: S 1.45918, E 5.64530 (sampled) and ‘Vite’: S 1.45904, E 5.62933 (observed)) from 10th–13th May, 2010 (Figure 2). Colony sizes were estimated independently by AJP and AFL. Bats were caught in a mist net (18m; 38mm) as they departed the roost site at dusk, or returned at dawn. Under manual restraint, up to 1 ml blood was collected from the propatagial vein using a citrated 1ml syringe and placed into a plain 1.5ml eppendorf tube. Morphometric and demographic details were recorded and a uniquely-numbered thumb ring was applied before the individual was released. Age class (sexually immature or adult) was assessed by body size and the degree of genital and nipple development. Blood samples were centrifuged immediately after morning sampling or allowed to settle overnight after night-time sampling. The plasma was aspirated and stored at −20°C. Samples were heat treated at 56°C for 30 min prior to analysis. Blood samples were collected from 75 bats, however five samples were of insufficient volume for full testing or were haemolysed. Consequently, serological analyses were conducted on 73 or 70 samples (henipaviruses and LBV respectively).

### Detection of antibodies against henipaviruses

Antibodies against henipaviruses (Hendra and Nipah viruses, HeV and NiV) were initially detected using Luminex multiplexed binding assays as previously described [18], [25]. Briefly, recombinant HeV and NiV glycoproteins were conjugated to internally coloured and distinguishable microspheres, allowing multiplexing. Antibody binding to each microsphere was detected after conjugation of bound antibodies with biotinylated Protein A and fluorescent streptavidin-R-phycoerythrin. Binding results are given as median fluorescence intensities (MFI) of at least 100 microspheres for each virus type. Samples were further analysed using Luminex inhibition assays and virus neutralisation tests (VNTs) as previously described [18], [25]. VNTs were undertaken at the Australian Animal Health Laboratory (AAHL).

Western blot analysis was performed on 11 sera with the highest MFI values in the binding assays (>750) using a purified recombinant NiV nucleocapsid protein [26]. Briefly, 50 µg of protein was electrophoresed on a 12% polyacrylamide gel. The protein was electroblotted overnight onto a nitrocellulose membrane which prior to this had been blocked in 5% skim milk powder (SMP) and subsequently cut into strips. The strips were then incubated for 1h with individual sera (diluted 1∶50 in 5% SMP). Polyclonal rabbit sera raised against the recombinant protein (1∶2000) as well as known NiV neutralising *Pteropus* bat sera (1∶50) were used as positive controls. Strips were washed and then incubated for 1h with Protein A/G conjugated to alkaline phosphatase (Thermo-Fisher Scientific Inc., USA). Strips were washed and then allowed to develop in the presence of alkaline phosphatase reagents for 15 minutes. The marker used was Benchmark prestained protein ladder (Invitrogen, UK).

### Detection of antibodies against Lagos bat virus

Antibodies against LBV (LBV.NIG56-RV1) were detected using a modified Fluorescent Antibody Virus Neutralisation (mFAVN) test, with positive and negative controls, as previously described [16], [27]. Confirmatory testing was undertaken on a subset of 2 positive and 16 negative mFAVN samples using a lentivirus pseudovirus neutralisation assay which had been previously validated against the mFAVN for *E. helvum* plasma [28]. Details of viruses, pseudotype production methods and assays are described elsewhere [28], [29]. In this study, assays were multiplexed with two viruses per assay LBV (as above) + Mokola virus (MOKV.NIG68-RV4) and Duvenhage virus (DUVV.RV131) + West Caucasian Bat Virus (WCBV) [28]. All samples were analysed in duplicate in both assays.

As in previous studies [16], [28], titres reported correspond to 100% neutralisation of pseudotype or virus input and are reported as IC_100_ endpoint reciprocal dilutions. Mean neutralising titres were considered positive at the next dilution level above that at which there was no neutralisation against rabies (CVS-11) in previous studies: >1∶40 for the pseudotype assay and >1∶9 for the mFAVN [16], [28].

### Statistical analyses

Linear regression models were implemented using the R package [30] to assess the comparative morphology of *E. helvum annobonensis* with *E. helvum* from continental populations and other islands (using data collected using the same methods by AJP, DTSH and KSB for other studies).

## Results

**Figure 3:**
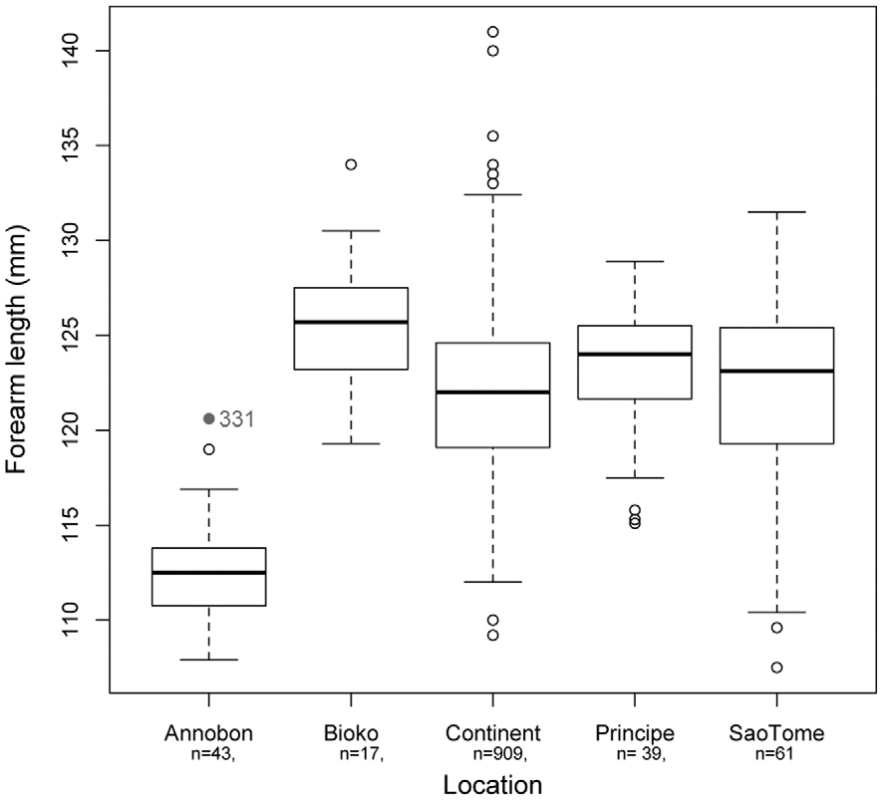
Forearm length of adult *Eidolon helvum* bats. Values are compared among populations in the four Gulf of Guinea islands and continental Africa. Graphs are of box and whisker plots showing median (black line), 25^th^ and 75^th^ percentile (box) and 1.5x the interquartile range (dotted line) values.

Colony sizes on Annobón were estimated at approximately 1000 – 1500 bats (sampled colony) and 600–1000 bats (observed colony). Captured individuals were classified as adult (n = 43, comprising 14 females and 29 males) or sexually immature (n = 32, comprising 19 females and 13 males) (Table 1). Most adult females (13/14) were in early stages of gestation, and based on a gestation period of 4 months [31], the birthing time was estimated as mid-September. Sexually immature individuals could be further classified based on size, weight and reproductive status into those which were estimated at 8 months old (n = 21) and those which were estimated at 20 months of age (n = 11). Adult body size and forearm length were significantly smaller (p<0.001) than *E. helvum* on either continental Africa or the other Gulf of Guinea islands (Figure 3).

**Table 1:** Age and gender classification for sampled *E. helvum annobonensis.*

Age	Male	Female	Total
Sexually immature: ∼8 months	10	11	21
Sexually immature: ∼20 months	3	8	11
Adult: 2+ years	29	14	43
Total	42	33	75

**Figure 4:**
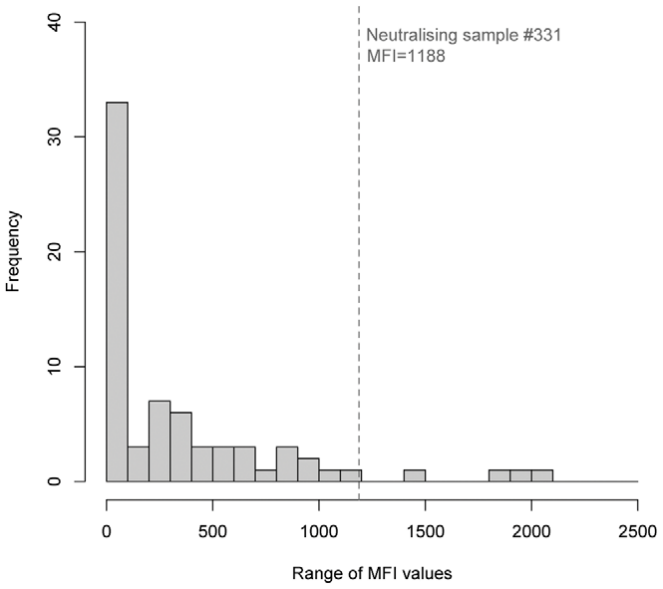
Frequency distribution of Luminex Median Fluorescent Intensity (MFI) values against NiV in *Eidolon helvum annobonensis*. The dotted line represents the where the MFI of the neutralising sample (Bat # 331) lies within this distribution.

**Figure 5:**
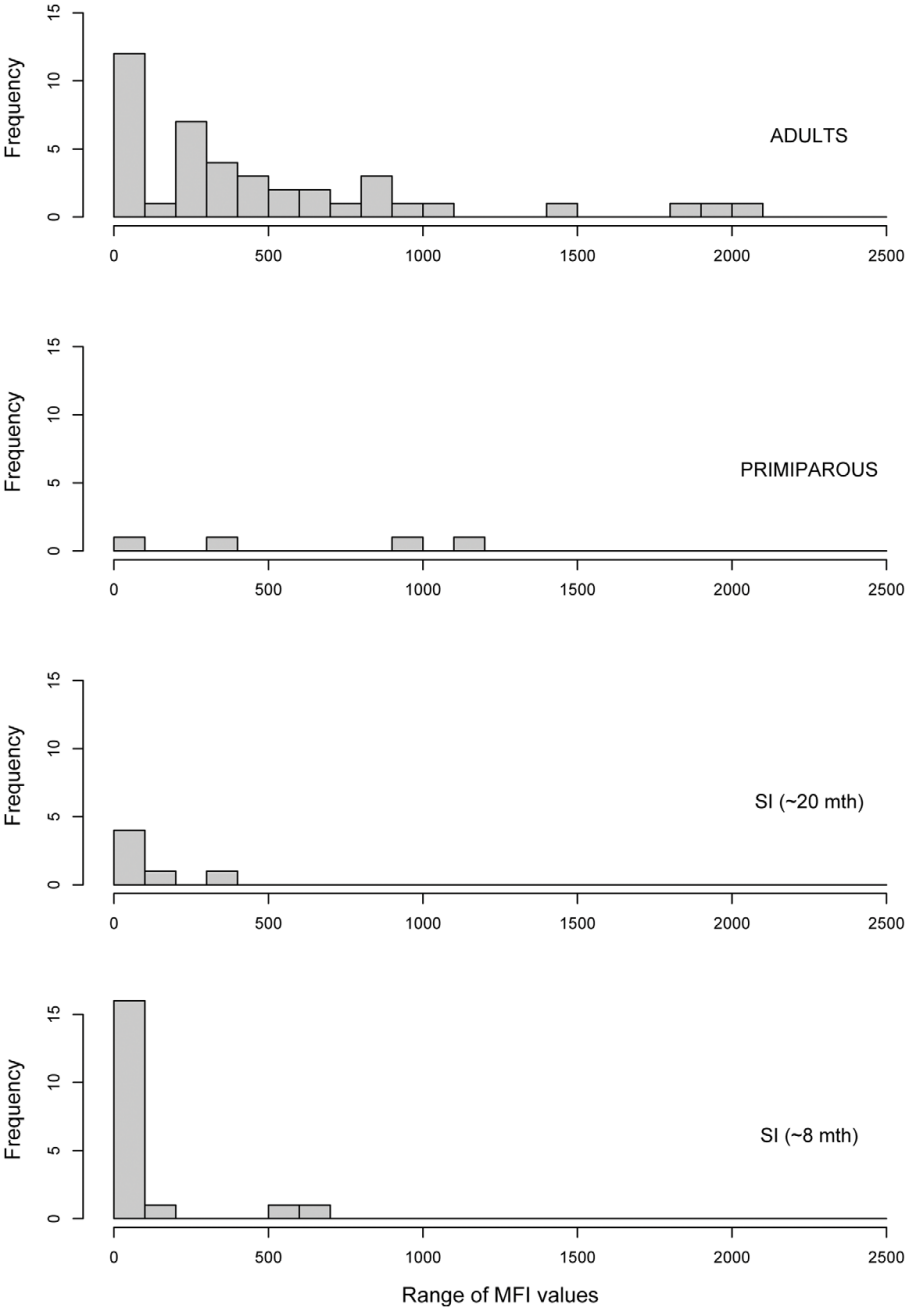
Frequency distribution of Luminex MFI values against NiV in *Eidolon helvum annobonensis*, separated by age.

**Figure 6:**
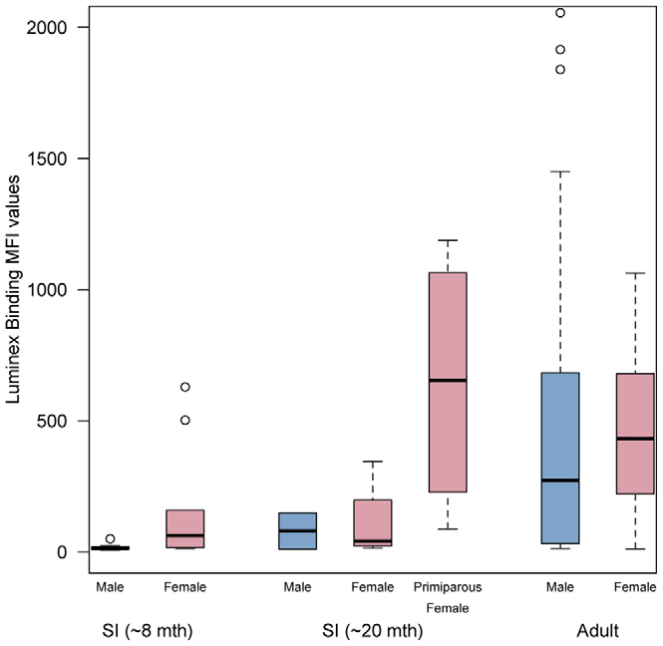
Luminex MFI values against NiV in *Eidolon helvum annobonensis*, separated by age and gender. Age is divided into bats of approx. 8 months of age, 20 months of age (including primiparous females) and adults. Graphs are of box and whisker plots showing median (black line), 25th and 75th percentile (box) and 1.5x the interquartile range (dotted line) values.

**Figure 7:**
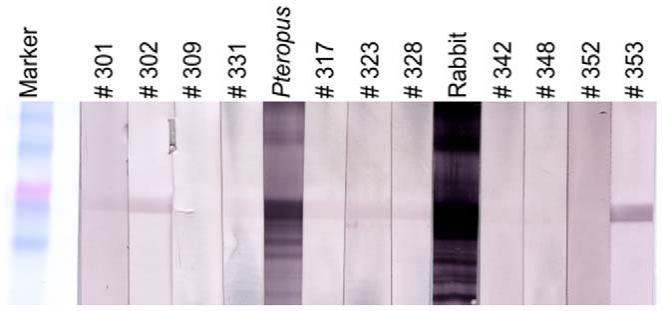
Results of western blot analysis. Samples with Luminex binding MFIs over 750 were tested using a recombinant, purified Nipah virus nucleocapsid protein. The marker is BenchMark Pre-stained Protein Ladder (Invitrogen); the positive control sera are NiV-neutralising *Pteropus alecto* and polyclonal Rabbit sera raised to recombinant NiV protein.

Consistent with previous studies in Ghana [18] and in continental Africa (unpublished data), Luminex binding MFIs were higher against NiV than HeV in all samples above background levels. We interpret this as a greater cross-reactivity (and presumably relatedness) between African henipaviruses (or henipavirus-like viruses) and NiV, than with HeV. For this reason, although the full set of results is presented in Table S1, the data discussed below refer to NiV only. In a previous study [18], three times the average MFI of negative bat sera was used as a threshold for positive reactivity for the binding assay and samples with an MFI>200 were considered positive. Here, 51% of Annobónese samples had MFI values<200, and the remaining samples ranged in MFI from 200–2055 (Figure 4). While adult individuals displayed a wide range of MFI values (6 – 2056), values for individuals from younger age classes were restricted within a lower range of MFIs. For example, 16/19 individuals in the youngest age class (8 months) had MFI values of <200, and the highest MFI was only 629 (Figure 5). A similar pattern was seen in the 20 month age class (including primiparous individuals), where 6/10 individuals had MFIs under 200, and the maximum was 1188 (Bat # 331). Luminex inhibition assay results were in agreement with binding results (Table S1). No significant differences in MFI values were observed between males and females overall, or between the sexes within age classes (p>0.05, Figure 6). Of 69 samples with sufficient plasma to enable testing by VNT, one sample (#331) neutralised HeV at 1∶40 dilution and NiV at 1∶80 dilution. Presence of antibodies was confirmed using western blot analyses (Figure 7) in 7 of 11 samples tested using this method. Reactivity to the recombinant NiV nucleocapsid protein, varied among positive samples from strong (#302, 353) to intermediate (#301, 331, 317, 323, 328). All bats with positive results in western blot analyses were adults, except for bat #331, a primiparous female.

Neutralising antibodies to LBV were detected at a dilution of >1∶9 in 1 of 72 samples (bat #352). This result was confirmed using the pseudotype assay, where neutralisation was observed at a dilution of >1∶80. An additional two samples showed weak neutralisation to LBV pseudotype particles (1∶20), and no samples neutralised WCBV, DUVV.131 or MOKV.NIG68-RV4.

## Discussion

Multiple studies have identified *E. helvum* as reservoirs of henipaviruses and lyssaviruses in continental Africa. After the original isolation of LBV in *E. helvum* in Nigeria [13], it has subsequently been isolated from this species in Senegal and Kenya [14], [15], and LBV antibodies have been reported in *E. helvum* populations in Ghana, Kenya and Nigeria [14], [16], [17]. Antibodies against henipaviruses and against henipavirus-like viral RNA have been detected in *E. helvum* in mainland Africa [12], [18]. This is outside the range of *Pteropus* fruit bats, the established reservoir hosts of henipaviruses [32]. HeV has been isolated from bats in Australia [32] and NiV from bats in south east Asia [33], however an African henipavirus has not yet been isolated. Our morphological analyses are consistent with earlier studies, highlighting the distinctiveness and isolation of *E. helvum annobonensis*. The results presented here raise intriguing questions on viral maintenance in small, isolated populations.

### Henipaviruses

In this study we demonstrated high MFIs in henipavirus Luminex binding assays and interpreted these as evidence of exposure of those individuals to henipa- or henipavirus-like viruses. Rather than presenting Luminex results as positive or negative, the MFI values are presented as we consider these to be more informative. The MFI values represent intensity of antibody binding to recombinant HeV and NiV G glycoproteins on a continuous scale. Usually, comparison of Luminex or ELISA results with a ‘gold standard’ assay such as a VNT through Receiver Operator Characteristic (ROC) curve analysis allows calculation of a threshold that determines the sensitivity and specificity of the screening assay. However, since no African henipavirus is available for neutralisation testing, no gold standard assay is available. Isolation of an African henipavirus and the development of virus-specific assays would be required to interpret our Luminex data in this traditional manner. Additionally, in a natural population where virus is circulating endemically, individual bat antibody levels are expected to be dynamic, reflecting factors such as time since last exposure and the total number of exposures over the individual's life span. Consequently, classifying titres as ‘positive’ or ‘negative’, while facilitating the calculation of population-level seroprevalences, overlooks the underlying dynamics of the system.

Based on henipavirus Luminex binding results, bats of 8 months of age demonstrated a very low range of MFI values compared with adult bats. Bats of 20 months of age also demonstrated a low range, except for two primiparous females with MFIs of 941 and 1188. Consistent with studies on other fruit bat species [34], [35], our continental studies have indicated that henipavirus maternal antibodies are present in *E. helvum* pups at birth at levels proportional to those in the dam, and wane over a period of 5–7 months (unpublished data). The majority of the most recent birth cohort in this study can therefore be inferred to be beyond the age at which maternal antibodies would be expected to have waned. The small numbers of individuals in the 8-month-old cohort with higher titres were assumed to have remnant low levels of maternal antibodies and hence would be fully susceptible to henipavirus infections once these titres had waned. The presence of high MFIs, positive western blot results and a seropositive VNT in older bats suggests that virus has circulated within the population within the last 20 months. More-detailed age-specific seroprevalences are required, however, to determine if this represents a single epidemic wave or ongoing endemic circulation. Bat #331, the only bat positive in all henipavirus assays, was classified as ‘sexually immature/primiparous’ due to a lack of developed nipples. Given the near 100% reproduction rate for adult females, this is usually sufficient for differentiating adult from sexually immature females. Interestingly, this bat's forearm length (120.6 mm) was significantly longer than all adults on Annobón, and within the range expected for bats from the continent and São Tomé (Figure 3).

It has been hypothesised that population-level persistence of henipaviruses in bats relies on a large, weakly-coupled, asynchronous metapopulation, and that populations can experience either acute ‘explosive’, or slow ‘smouldering’ epidemics as a result of spatial heterogeneity in population herd immunity [36]. An ongoing supply of susceptible individuals for new infections via movement among subpopulations or seasonal demographic changes ensures metapopulation-level persistence. The CCS for henipavirus persistence in bat populations is unknown, however in other species, the CCS for other paramyxoviruses is in the order of hundreds of thousands or more individuals [37], [38]. Given the isolation of the fruit bat population on Annobón, it was expected that the estimated total population size of 1600–2500 individuals on the island would be too small to allow the persistence of henipaviruses. Our results indicate, however, that the fruit bats on Annobón have previously been infected with a virus, or viruses, which are serologically cross-reactive with HeV and NiV. Given the low level of *E. helvum* gene flow to Annobón, it is unlikely that an infectious immigrant arrived within the 20 months prior to sampling; however, the presence of a seropositive primiparous female which is significantly larger than all other adult bats opens up this possibility, and indicates a potential mechanism for the presence of henipavirus on Annobón.

An alternative hypothesis is that there is persistence of henipavirus infection within individuals with recrudescence, such as during times of stress or breeding. The related HeV in Australia has the ability to persist and fatally recrudesce in both horses [39] and humans [40]. NiV has also been shown to recrudesce as encephalitis in humans from several months to as long as 4 years after the initial infection [41], and could also have this ability in bats in Malaysia [34], [42], but population-level studies are lacking. Whilst henipavirus antibodies and virus have been detected in island fruit bats in Asia [33], [43], [44], study species were either in contact with migratory species or of sufficient proximity to the mainland or larger island populations that they cannot be considered isolated from the metapopulation as a whole [45], [46]. The presence of previously-infected bats in the very small population on the most isolated island, Annobón, may provide evidence from wild bat populations for viral persistence within individuals, with recrudescence as a mechanism for population-level persistence. More information on, for example, henipavirus pathogenesis in *E. helvum*, within-host viral dynamics and immune responses to henipaviruses is needed to clarify this. Longitudinal serological surveys of the bats on Annobón would enable further interpretation of data and the investigation of factors currently hypothesised as important for virus persistence on the island.

It is unclear how soon *E. helvum* arrived on Annobón island after it was formed 4.8 Mya. No known records exist as to whether *E. helvum* bats were present on the islands at the time of Portuguese colonisation in the late 15th century, however the degree of genetic and morphological differentiation present in *E. helvum annobonensis* is indicative of independent colonisation by the bats prior to this time. In the absence of viral sequences from Annobón, it is not possible to conclude whether henipa- or henipa-like viruses demonstrated here were introduced to Annobón at the time of *E. helvum* colonisation, or by rare dispersal events. Support for the former lies in the long evolutionary history between fruit bats and henipaviruses [47], [48], but is dependent on whether future experimental studies are able to demonstrate further evidence for individual-level persistence and recrudescence as a mechanism for population-level persistence.

The presence of other bat species might also contribute to virus persistence on Annobón. There is only one record of another bat species being present on the island (the Mauritian tomb bat, *Taphozous mauritianus*), a species which has a widespread distribution across Africa, similar to that of *E. helvum* [49]. However, *T. mauritianus* has not been observed in subsequent surveys in Annobón, and if it is still present on the island, appears extremely unlikely to be contributing to henipavirus persistence in *E. helvum annobonensis*.

### LBV

One adult male bat (# 352) demonstrated neutralising antibodies to LBV using the gold standard mFAVN and a validated lentiviral pseudotype assay. This observation adds to the numerous reports of the presence of lyssavirus neutralising antibodies in otherwise-healthy bats (as reviewed in [50]). In contrast to bat #331, there was nothing about this individual's morphology to suggest that it may have been an immigrant. With a single positive individual, evidence for the presence of LBV in this isolated population of *E. helvum annobonensis* is unclear, and the contrast with reports of LBV seroprevalences of circa 40% in *E. helvum* populations in other regions of Africa is marked. These data suggest that this population of *E. helvum annobonensis* is refractory to LBV infection, that mixing of *E. helvum annobonensis* with mainland *E. helvum* is sufficiently rare to prevent inter-population transmission of LBV, or that the population is too small to support persistent transmission.

While lyssavirus prevalence is usually low in bat populations (<4%), seroprevalence is often much higher (e.g. up to 70% for rabies in Brazilian free-tailed bats, *Tadarida brasiliensis* [51]–[53], and 14 – 44% (Nigeria), 37% (Ghana), and 40–67% (Kenya) for LBV in *E. helvum* [14], [16], [17]. In contrast, studies on European bat lyssavirus 2 (EBLV2) have detected low seroprevalences (1–4%) in Daubenton's bats (*Myotis daubentonii*) in the United Kingdom [54]. Rabies virus has been shown to persist in populations of temperate insectivorous bats as a result of a long incubation period and lowered mortality and transmission during the hibernation period [55], [56]. A long incubation period has been hypothesised to facilitate viral persistence in migratory bats, such as mainland *E. helvum* [50]. The extended incubation periods known to exist for lyssaviruses may provide a mechanism that allows LBV persistence within isolated populations, by avoiding fade-out before new susceptibles are provided by the seasonal birth pulse. Natural and experimental studies on lyssavirus transmission, pathogenesis and serological response in bats, however, have produced highly variable intra- and inter-study results (as reviewed by [50]) and further studies are required.

### Implications

Here, we demonstrate of the presence of neutralising antibodies using multiple HeV, NiV and LBV assays within an isolated population of bats, providing evidence of exposure of individuals in this population to these, or closely-related, viruses. However, inferring the viral *dynamics* from a cross-sectional sampling event (such as the one described here) is problematic and longitudinal sampling is required to make such inferences. The rate of decay of antibodies to henipaviruses or LBV in naturally-infected bats has not been fully elucidated, however, one study indicates that NiV antibodies may persist in individual adult bats for at least 14 months, whereas juvenile antibody levels wane over a period of up to 7 months [34]. Rabies virus neutralising antibodies were shown to wane in experimentally-infected bats within 6 months after an initial inoculation, but persisted for longer (6–12 months) after a second inoculation of surviving bats [57].

*E. helvum* is known for its close contact with human populations in continental Africa [58], and this is also the case on Annobón, where bats and pigs feed on fruit trees within the main town, Palé. A recent study by our group demonstrated antibodies against henipaviruses in a sample of domestic pigs in Ghana [59]. Our results, therefore, could have important public health implications, but more information is required on the viruses involved, their infection dynamics within the bat populations, potential spillover routes, and bat population dynamics before any risk can be assessed. Challenging transport and working conditions in this remote setting precluded the gathering of reliable viral molecular data; this will be the focus of future sampling trips.

### Conclusions

Isolated island populations, such as *E. helvum annobonensis* in the Gulf of Guinea, present a unique and valuable opportunity to further our understanding of the maintenance of viruses in wildlife populations. Whilst cross-sectional serological studies cannot provide details on viral dynamics within populations, valuable information on the presence or absence of virus infections may be obtained. Further studies are required to bring anecdotal theory and empirical data together to understand fully how viruses which are considered to be acute and immunising may be maintained in small populations.

## Supporting Information

Table S1 **Sample details and serology results for** ***E. helvum annobonensis*** **.** Empty cell indicates sample not tested. ^*^ SI.1: Sexually immature individual estimated at 8 months old; SI.2: Sexually immature male or non-pregnant female individual estimated at 20 months old; PPA: Periparturient female, estimated at 20 months old; A: Adult.
(XLS)
